# Enhancing the quality and lipid stability of chicken nuggets using natural antioxidants

**DOI:** 10.1186/s12944-017-0496-4

**Published:** 2017-06-08

**Authors:** Muhammad Sajid Arshad, Ali Imran, Muhammad Tahir Nadeem, Muhammad Sohaib, Farhan Saeed, Faqir Muhammad Anjum, Joong-Ho Kwon, Shahzad Hussain

**Affiliations:** 1 Institute of Home and Food Sciences, Government College University Faisalabad, Punjab, Pakistan; 2 School of Food Science and Biotechnology, Kyungpook National University, Daegu, South Korea; 3 Department of Food Science and Human Nutrition, University of Veterinary and Animal Sciences, Lahore, Pakistan; 4 College of Food and Agricultural Sciences, King Saud University, Riyadh, Saudi Arabia

**Keywords:** Wheat germ oil, Fatty acid, α-lipoic acid, Sensory evaluation, Chicken nuggets

## Abstract

**Background:**

Current day consumers prefer natural antioxidants to synthetic antioxidants because they are more active. However, the activity generally depends on the specific condition and composition of food. The aim of this study was to investigate the effect of wheat germ oil and α-lipoic acid on the quality characteristics, antioxidant status, fatty acid profile, and sensory attributes of chicken nuggets.

**Methods:**

Six types of diets were prepared for feeding the chickens to evaluate the quality of nuggets made from the leg meat of these experimental animals. These included control, diet enriched with wheat germ oil (WGO), which is a rich natural source of α-tocopherol (AT), diet with added AT or α-lipoic acid (ALA), diet with a combination of either ALA and WGO (ALA + WGO) or ALA and synthetic AT (ALA + AT). ALA has great synergism with synthetic as well as natural AT (WGO).

**Results:**

The diet with WGO and ALA showed the best potential with respect to both antioxidant activity and total phenolic content. HPLC results revealed that the chicken nuggets made from WGO + ALA group showed maximum deposition of AT and ALA. The stability of the nuggets from control group was found to be significantly lower than that of nuggets from the WGO + ALA group. Total fatty acid content too was higher in the nuggets from this group. The poly unsaturated fatty acids (PUFA) were found to be higher in the nuggets from the groups fed with a combination of natural and synthetic antioxidants.

**Conclusion:**

It is concluded that the combination of natural and synthetic antioxidants in the animal feed exerts a synergistic effect in enhancing the stability and quality of chicken nuggets.

## Background

Using synthetic or natural antioxidants is a major strategy used to prevent lipid oxidation. The current trend in the food industry is using natural additives in preference to synthetic additives [1]. The growth of the animals and the antioxidant potential of the meat from these animals can be improved by the addition of antioxidants as dietary supplements to animal feed. The addition of natural antioxidants to animal feed enhances the lipid stability, improves the sensory attributes of meat, and improves the functionality of meat products [2, 3].

The quality of meat increased by the addition of antioxidants in the animal feed [4]. Different types of antioxidants are added to the animal feeds for different purposes. The use of synthetic and natural antioxidants has synergistic effect on the quality as well as the antioxidant potential of meat. Wheat germ and wheat germ oil (WGO) have been used as dietary supplements for different purposes [5]. Wheat germ contains mainly protein (26–35%), lipids (10–15%), and minerals (4%) and major bioactive compounds like tocopherol, policosanols, and sterols [6]. Wheat germ is the richest plant source of vitamin E. The meat quality from chickens can be improved by dietary supplementation of vitamin E, which alleviates oxidative stress [7].

WGO is also rich in essential fatty acids like linoleic acid and alpha linolenic acid. These are helpful in performing numerous functions like lowering the cholesterol level, and enhancing endurance. WGO also stimulates the tocopherol redox-system by changing the lipid peroxidation [8]. In addition to vitamin E and essential fatty acids, WGO also contain Vitamin B complex and is important for chemoprevention [8]. WGO, because of its vitamin E content, protects cells against free radicals, which negatively impact the metabolism.

ALA, widely distributed in many foods, is a short chain fatty acid and is a powerful natural antioxidant [9]. ALA is not only an antioxidant but also a co-factor for many enzymes. ALA stimulates glucose oxidation in muscles and improves insulin sensitivity. It is also helpful to reduce the oxidative stress in tissues of different mammals [10]. The antioxidant potential of both ALA and AT is helpful in reducing lipid oxidation in both raw and processed chicken meat and meat products [11, 12]. Antioxidants in chicken meat also reduce drip loss by lowering the post mortem pH of the meat [13].

The aim of the present project was to investigate the antioxidant potential of different natural antioxidants and their effect on the stability, sensory attributes, and fatty acid profile of chicken nuggets made from the meat of animals fed with different antioxidants individually or in combination.

## Methods

### Procurement of raw materials

All the chemicals and reagents required for the study were purchased from Sigma Aldrich (Tokyo, Japan) and Merck (Merck KGaA, Darmstadt, Germany). This research project was conducted at the National Institute of Food Science and Technology (NIFSAT) and Nutrition Research Center, University of Agriculture, Faisalabad, Pakistan. The experimental design included 6 different diets supplemented with various antioxidants, singly or in combination; control, WGO, AT, ALA, WGO + ALA and AT + ALA. Chicken nuggets were made from the leg portion of chickens fed with these diets.

### Chicken nugget preparation and processing

Antioxidant enriched chicken meat was used for the preparation of nuggets by following the method described by Perlo et al. [14]. The nuggets were stored for 45 days at −18 °C and analysis was done at 15-day intervals. The raw material for manufacturing the nuggets was weighed and cleaned according to the recipe. The recipe for the preparation of nuggets is as follows. Boneless chicken (500 g), Egg (1), oil (as required for frying), black pepper (12 g), garlic paste (1 tsp), onion (1), plain flour (120 g), bread crumbs (70 g), and salt (20 g). The control and antioxidant enriched broiler meat (leg) were washed multiple times with tap water, deboned manually and minced using an electric mincer to very fine consistency for preparing nuggets of excellent texture. The minced meat and onions were mixed in a meat mixer for 5 min, followed by the addition of all other ingredients according to the recipe and mixed using a meat mixer to obtain a uniform blend. When all the ingredients were thoroughly mixed, the mixture was spread into a thin layer (10 mm thickness) and shaped into discs of 30 mm diameter (10 ± 1 g/piece). The nuggets were dipped sequentially in plain flour and bread crumbs and fried in canola oil at 180 °C till golden brown in color.

### Antioxidant status of chicken nuggets

The antioxidant status of the chicken nuggets was determined by different methods described below. The total phenolic content (TPC) in the nuggets was determined by adopting the procedure described by Senevirathne et al. [15]. The total phenolic content of the nuggets was estimated as gallic acid equivalent (mg GAE/100 g). The nugget samples were subjected to 2,2- diphenyl-1-picrylhydrazyl (DPPH) radical scavenging activity analysis according to the procedure outlined by Brand-Williams et al. [16]. Percentage neutralization of free radicals was assayed using DPPH and calculated using the following formula. % Neutralization = 100 × (A_blank_- A_sample_/ A_blank_). ABTS^+^ reducing activity of the nugget samples was measured using the method described by Erel [17]. ABTS^+^ reducing activity (%) = [(A_control_ – A_sample_) ÷ A_control_] × 100. The ferric reducing antioxidant power (FRAP) in the nugget samples was estimated using the method described by Arshad et al. [18] with some modification.

### Physico-chemical analysis of nuggets

pH of the nuggets was measured with a pH meter according to the method described by Sallam et al. [19]. Ten grams of the sample was homogenized with 50 mL distilled water and pH was measured using a digital pH meter. A hand-held tristimulus colorimeter (Color Test Meter II) was used to determine the color of the nuggets at regular storage intervals (0, 15, 30, and 45 days) by following the procedure described by Elgasim and Al-Wesali [20]. Color was determined by placing the nuggets in a petri plate under a photocell. The water content of the nuggets was measured using electronic Hygropalm water activity meter (Model Aw-Win, Rotronic, equipped with a Karl-Fast probe) at regular storage intervals, using the method described by Cosenza et al. [21]. The textural characteristics of nuggets were measured at different storage intervals by using a texture analyzer (Mod. TA-XT2, Stable Microsystems, surrey, UK) as described by Cardoso et al. [22]. The nuggets were fried and compression test was performed to check the texture of the product.

### Lipid stability analysis

The lipid stability of chicken nuggets was determined in terms of the amounts of thiobarbituric acid-reactive substances (TBARS) and peroxide value (POV). The amount of TBARS in the nuggets was estimated as per the guidelines of Liu et al. [23] and TBARS was expressed as milligrams of malondialdehyde (MDA)/kg meat. The POV of the nuggets was determined by the method outlined by Shantha and Decker [24] and expressed as meq peroxide/kg.

### Quantification of α-lipoic acid and α-tocopherol

The ALA content in the nugget samples was measured according to the method described by Satoh et al. [25] with some modifications. The nugget samples were prepared according to the method described by Asghar et al. [26] for the determination of AT. HPLC chromatograms were obtained by using a C18 column, (250 mm × 4.6 mm, 5.0 μm), System controller SCL-10 A, water pump LC-10 AT, and flow controller valve FCV-10 AL with a mobile phase of 100% methanol at a flow rate of 1 mL/min.

### Fatty acid Profile

Total fatty acids were extracted from nugget samples per the method described by Folch et al. [27]; this method uses an antioxidant to prevent oxidation during sample preparation and a flame ionization detector (FID). The injector temperature was 250 °C and the detector temperature was 300 °C. The column temperature program initiated the runs at 100 °C for 2 min, warmed to 170 °C at 10 °C /min, held for 2 min, warmed to 220 °C at 7.5 °C /min and then again held for 20 min to facilitate optimal separation. Results are presented as percentage of total fatty acids.

### Sensory evaluation

Sensory evaluation of the nuggets was carried out by a trained panel at different storage intervals (0, 15, 30, and 45 days), using a 9-point hedonic scale (9 = like extremely; 1 = dislike extremely), as per the guidelines of Meilgaard et al. [28]. Sensory assessment for various quality attributes of nuggets, such as appearance, flavor, taste, and overall acceptability, were recorded. All evaluations were conducted by panelists trained in sensory evaluation of foods made from muscle meat. The panelists carried out the evaluation in individual booths under clear white fluorescent light in the Sensory Evaluation Laboratory of NIFSAT, University of Agriculture, Faisalabad, Pakistan. During the evaluation process, the panelists were provided unsalted crackers, mineral water, and expectorant cups to neutralize and rinse their taste receptors between different samples to facilitate rational assessment. The descriptors were rated on a scale from “0” representing the lowest score and “9” the highest. The panelists were requested to rate the product quality by scoring for the selected parameters.

### Statistical analysis

The work was carried out using completely randomized design (CRD), and the data obtained for different parameters was analyzed statistically using the Statistical Package, Statistic 8.1. Levels of significance (*P* ≤ 0.05) were determined (ANOVA) using 2-factor factorial CRD by following the principles outlined by Steel and Torrie [29]. The means were compared using LSD.

## Results and Discussions

### Antioxidant potential of chicken nuggets

**Fig. 1:**
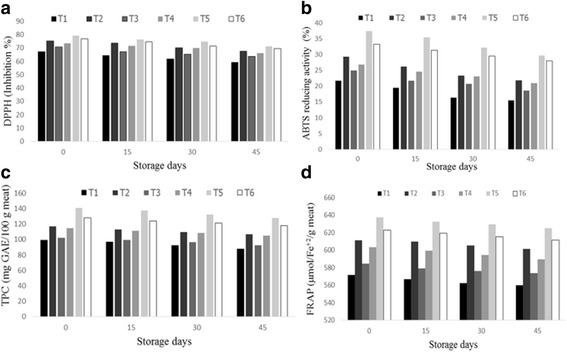
(**a-d**): (**a**) DPPH free radical scavenging activity of the chicken nuggets (**b**) ABTS reducing activity of the nuggets (**c**) Total phenolic contents of the nuggets (**d**) FRAP of the nuggets

The stable nitrogen radical having yellow color is a DPPH radical and it can be easily solubilized in organic solvents [30]. The metastable radical having blue color is the ABTS radical and dissolves easily in water as well as organic solvents [31]. The results showed that the nuggets from different diet fed animals had significant antioxidant parameters like DPPH, FRAP, ABTS and TPC at all storage intervals as shown in Fig. 1. The group containing a combination of ALA and WGO showed maximum percentage inhibition DPPH (79.24%) and ABTS (37.44%), whereas the percentage inhibition DPPH (67.35%) and ABTS (21.67%) were lowest in control group at 0 day of storage. The DPPH free radical scavenging activity and ABTS decreased as the storage duration increased. The group having WGO + ALA displayed higher free radical scavenging activity (71.26%) and ABTS (29.65%) on the 45th day of storage compared to that of control (59.37% and 15.48%). The results showed that the antioxidant activity of chicken nuggets was more in the group containing natural antioxidants compared to that of other groups. These results are in accordance with the findings of Arshad et al. [18, 32] who speculated that the antioxidant activity of raw and cooked chicken meat was higher when the animals were fed with a diet containing antioxidants that act synergistically. The results are further supported by Jung et al. [33] and Selim et al. [34] who showed that chicken meat from animals fed with dietary natural antioxidants has more antioxidant activity compared to that from control diet fed animals.

The group with combination of ALA and WGO showed maximum antioxidant power (637.56 μmol/Fe^+2^/g meat) and TPC (140.92 mg GAE/100 g meat) compared to those in the control (571.93 μmol/Fe^+2^/g meat) and TPC (99.02 mg GAE/100 g meat) at 0 day of storage. The ferric reducing antioxidant power and TPC decreased as the storage intervals increased. FRAP (624.84 μmol/Fe^+2^/g meat) and TPC (87.62 mg GAE/100 g meat) were higher at 45th day of storage in nuggets from the group fed with WGO + ALA, whereas the values were lower in control group (559.67 μmol/Fe^+2^/g meat and 87.62 mg GAE/100 g meat). Different studies proved that there was positive correlation between the antioxidant activities and total phenolic contents in raw and cooked chicken meat [11, 35–37]. The results showed that the antioxidant potential was higher in the meat from the group fed with both natural antioxidant (WGO) and ALA. This finding is consistent with those of Mancini et al. [38], who speculated that by using antioxidants like turmeric and ascorbic acid, the antioxidant potential (DPPH, ABTS and FRAP) in rabbit burgers could be increased compared to that of the control where no antioxidants were used during storage. Banerjee et al. [39] showed that goat meat enriched with natural antioxidants showed higher FRAP and DPPH values than those of the control.

### Physico-chemical properties of chicken nuggets

One of the important physical factors in the postmortem stage is pH. Many other factors such as cooking loss and color also affect the different sensory parameters [40]. Our results showed that different diets and storage intervals had significant effect on the pH and color of the nuggets as shown in Table 1. The results showed that the nuggets from the group which had the combination of ALA and WGO had significantly higher pH (6.47) and color (102 CTn) on the 45th day of storage, whereas those from control group of animals had the lowest pH (5.86) and color (92 CTn). pH showed a trend of increasing with increasing duration of storage. Our results are consistent with those of Chidanandaiah et al. [41], who reported that the pH of buffalo meat patties increased with the duration of storage. Jay [42] reported that the increase in pH during storage might be due to the fact that on meat and meat products, there is an accumulation of metabolites of bacterial action and deamination of meat proteins. The results are further supported by Sureshkumar et al. [43] who found that pH of buffalo meat sausages increased on storage and Kumar and Tanwar [44] who reported an increase in the pH of chicken nuggets on storage. The color value of chicken nuggets decreased during storage while higher color value was observed in groups benefiting from the synergism of antioxidants. The results are in agreement with the findings of Chandralekha et al. [45] who reported that the color value of chicken meat balls supplemented with natural antioxidants was higher than that of the groups receiving other treatments. They also reported a decrease in the color value during storage, which was a result of oxidation. These results were further supported by the study of Kala et al. [46] who showed that the color value of chicken patties decreased during storage, but increased when they contained antioxidants. Arshad et al. [32] recently confirmed that the pH value of meat increased and color value decreased with storage when there was no antioxidants supplemented in the feed, but increased with storage when enriched with antioxidants.

**Table 1:** pH and color of the chicken leg meat nuggets

Treatments	pH of leg nuggets with storage days					Color of leg nuggets with storage days				
	0	15	30	45	Mean	0	15	30	45	Mean
Control	5.76	5.8	5.83	5.86	5.81e	104	100	96	92	98e
WGO	5.92	5.96	6	6.03	5.98c	112	107	102	99	105b
AT	5.84	5.86	5.91	5.94	5.89d	107	102	98	94	100d
ALA	5.92	5.94	5.98	6.05	5.97c	110	104	101	98	103c
WGO + ALA	6.34	6.38	6.42	6.47	6.4a	116	110	106	102	109a
WGO + AT	6.08	6.1	6.16	6.21	6.14b	113	108	103	100	106b
Mean	5.98d	6.01c	6.05b	6.09a		110a	105b	101c	98d	

The results are mean of three independent measurements. Means sharing of the similar letter are statistically non-significant (*P* > 0.05) in a row or in a column

Control, WGO = (wheat germ oil (Natural α-tocopherol) 200 mg/ kg feed), AT (α-tocopherol 200 mg/kg feed), ALA = α-Lipoic acid 150 mg/kg feed, WGO + ALA = wheat germ oil (Natural α-tocopherol) 200 mg/ kg feed) + α-Lipoic acid 150 mg, ALA + AT = α-Lipoic acid 150 mg + α-tocopherol 200 mg/ kg feed

Water activity and texture values of the chicken nuggets are presented in Table 2. These results showed that the water activity and texture of the nuggets varied significantly with different diets and storage intervals. It is evident from Table 2 that the group fed with the combination of ALA and WGO showed significantly higher water activity (0.863) and lower texture value (1119) than those of the control (water activity: 0.813 and texture: 1139) on 0 day of storage. On the 45th day of storage, the group that was fed with the combination of ALA and WGO depicted higher water activity (0.783) and lowest texture value (1131), compared to the control group (water activity: 0.737 and texture: 1154).

**Table 2:** Water activity and texture of the leg chicken nuggets during storage

Treatments	Water activity					Texture				
	0	15	30	45	Mean	0	15	30	45	Mean
Control	0.813	0.787	0.763	0.737	0.775d	1139	1144	1150	1154	1147a
WGO	0.843	0.813	0.793	0.763	0.803c	1129	1134	1137	1143	1136c
AT	0.833	0.803	0.78	0.753	0.793 cd	1139	1140	1146	1150	1144b
ALA	0.84	0.81	0.79	0.76	0.8c	1133	1135	1139	1145	1138bc
WGO + ALA	0.863	0.84	0.82	0.783	0.827a	1119	1123	1127	1131	1125d
WGO + AT	0.853	0.823	0.803	0.773	0.813b	1125	1131	1136	1141	1133c
Mean	0.841a	0.813b	0.792c	0.762d		1131d	1135c	1139b	1144a	

The results are mean of three independent measurements. Means sharing of the similar letter are statistically non-significant (*P* > 0.05) in a row or in a column

Control, WGO = (wheat germ oil (Natural α-tocopherol) 200 mg/ kg feed), AT (α-tocopherol 200 mg/kg feed), ALA = α-Lipoic acid 150 mg/kg feed, WGO + ALA = wheat germ oil (Natural α-tocopherol) 200 mg/ kg feed) + α-Lipoic acid 150 mg, ALA + AT = α-Lipoic acid 150 mg + α-tocopherol 200 mg/ kg feed

The results showed that the water activity in chicken nuggets from the control decreased whereas the shear force value for texture increased during storage. There was increased water activity in chicken nuggets from the group fed with antioxidants. Sohaib et al. [11] reported that the water activity was higher and shear force value for texture was lower in antioxidant enriched chicken nuggets. Water activity tended to decrease and texture value tended to increase with storage. These results were also supported by the Arshad et al. [32]. Malav et al. [47] speculated that the decrease in shear force value observed in mutton patties enriched with antioxidant was due to the reduction in compactness because of the higher moisture content and aeration that lowers the shear force. The shear force value increased during storage because of the myofibrillar protein oxidation and the resultant increase in cross-linking and aggregation in meat [48, 49].

### Thiobarbituric acid reactive substances and peroxide value of the chicken nuggets

Lipid oxidation is a vital phenomenon that determines the quality of meat and meat products because of its effect on protein oxidation and discoloration of the meat. Lipid oxidation during storage is also the cause of rancidity and the consequent bad odor in meat and meat products [50]. The results of the lipid oxidation analysis by using the TBARS and POV parameters are shown in Table 3. TBARS and POV of the nuggets varied significantly with the type of diet and duration of storage. Higher TBARS (0.55 mg MDA/kg) and POV (0.91 meq peroxide/kg) values were observed on the 45th day of storage in nuggets from control of animals, which were fed basal diet. TBARS (0.37 mg MDA/kg) and POV (0.74 meq peroxide/kg) values of the nuggets were lower when the diet of the chickens was supplemented with the combination of ALA and WGO. This is in agreement with the findings of Al-Hijazeen et al. [50] who reported that the meat from chickens fed with antioxidant supplemented diet showed reduced TBARS value compared to that from control. They also speculated that there was greater increase in TBARS values during storage in cooked meat than in the raw meat. Arshad et al. [18] also reported that raw chicken meat displayed lower TBARS value compared to that of the chicken nuggets made from animals fed with the same diet. Khan et al. [12] reported that the nuggets from chickens fed diet rich in ALA and AT showed lower POV as compared to nuggets from control, which is supported by the results of our present study, where nuggets from WGO and ALA enriched group had minimum POV compared to those from other groups. Furthermore, different researchers speculated that the chicken breast nuggets supplemented with natural and synthetic antioxidants showed the lowest TBARS value, which supports the findings of the current study [3, 32].

**Table 3:** Thiobarbituric acid reactive substances and peroxide value of the leg chicken nuggets during storage

Treatments	TBARS (mg MDA/kg)					POV (meq peroxide/kg)				
	0	15	30	45	Mean	0	15	30	45	Mean
Control	0.37	0.43	0.48	0.55	0.46a	0.75	0.79	0.85	0.91	0.83a
WGO	0.27	0.31	0.36	0.41	0.34c	0.63	0.68	0.74	0.78	0.71c
AT	0.31	0.36	0.41	0.45	0.38b	0.68	0.72	0.76	0.82	0.75b
ALA	0.28	0.33	0.37	0.42	0.35c	0.64	0.70	0.75	0.80	0.72c
WGO + ALA	0.22	0.27	0.32	0.37	0.30e	0.59	0.64	0.68	0.74	0.66d
WGO + AT	0.25	0.29	0.34	0.4	0.32d	0.61	0.67	0.71	0.78	0.69 cd
Mean	0.28d	0.33c	0.38b	0.43a		0.65d	0.70c	0.75b	0.81a	

The results are mean of three independent measurements. Means sharing of the similar letter are statistically non-significant (*P* > 0.05) in a row or in a column

Control, WGO = (wheat germ oil (Natural α-tocopherol) 200 mg/ kg feed), AT (α-tocopherol 200 mg/kg feed), ALA = α-Lipoic acid 150 mg/kg feed, WGO + ALA = wheat germ oil (Natural α-tocopherol) 200 mg/ kg feed) + α-Lipoic acid 150 mg, ALA + AT = α-Lipoic acid 150 mg + α-tocopherol 200 mg/ kg feed

### α-Lipoic acid and α-tocopherol contents

**Fig. 2:**
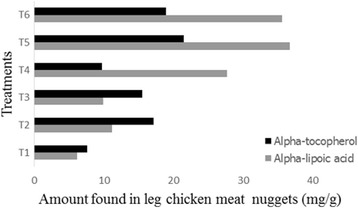
Alpha-tocopherol and α-lipoic acid content (mg/g) in the nuggets

ALA is both fat and water soluble in nature, scavenges the free radicals and also has the ability to regenerate other antioxidants like AT and ascorbic acid. ALA also acts as a coenzyme in different pathways of carbohydrate metabolism [9, 35]. The results of the quantification of ALA and AT by HPLC in leg nuggets are shown in Fig. 2. These results showed that the content of ALA and AT varied significantly with the type of diet. As evident from Fig. 2, the group that benefited from the synergism between WGO and ALA showed the best results not only for ALA (36.77 mg/g) but also for AT (21.47 mg/g). Nuggets from the group that was fed only basal diet contained much lower quantities of both ALA (6.14 mg/g) and AT (7.63 mg/g). The results are in agreement with the findings of Yasin et al. [36] and Parveen et al. [37] who speculated that the chickens whose diet was supplemented with higher quantities of ALA (150 mg/Kg) showed higher content of ALA and AT deposited in the raw meat. Khan et al. [12] also supported the idea of the synergism between ALA and AT because ALA regenerates some other antioxidants as mentioned above.

### Fatty acid profile of chicken leg nuggets

The contents of individual fatty acids, saturated fatty acids (SFA), monounsaturated fatty acids (MUFA), polyunsaturated fatty acids (PUFA), unsaturated fatty acid (UFA) and the ratios of SFA/UFA and PUFA/SFA in chicken nuggets were affected by different diets as shown in Table 4. A total of 9 fatty acids were detected in the nuggets. Oleic acid (C_18:1_) was found to be in higher quantities compared to all the other fatty acids. The content of oleic acid was significantly higher (35.29%) in the nuggets from chickens fed with WGO supplemented diet and lowest (32.55%) was found in group where combination of AT and ALA given to the diet of chicken. Margaric acid (C_17:0_) was present in the lowest quantity compared to all the other fatty acids. Palmitic acid (C_16:0_) was found to be the next highest after oleic acid and its content ranged from 17.11 to 22.34%. The MUFA (38.23%) and PUFA (24.31%) were also higher in the nuggets from the group fed with WGO and lowest in the nuggets from the group fed with WGO + AT. The ratio of PUFA and SFA is also very important and this ratio ranged from 0.65 to 0.69 in the nuggets made from the groups fed with different diets with no significant difference among the different groups. Our results are in agreement with the outcomes of Nkukwana et al. [51], who reported that there was no significant difference in the ratio of PUFA to SFA, which ranged from 0.68 to 0.82 in the meats of the animals receiving different antioxidant treatments. The recommended ratio for PUFA/SFA was around 4 [52]. Significantly higher PUFA/SFA ratio was found in broiler meat of animals fed with plant-based diet and showed higher PUFA levels [53]. The results were further supported by Arshad et al. [54] who speculated that the content of polyunsaturated fatty acids was higher in groups where the diet was supplemented with WGO.

**Table 4:** Fatty acid composition and profile in chicken nuggets

		Control	WGO	AT	ALA	WGO + ALA	WGO + AT
fatty acids (%) in leg chicken nuggets	^1^C_16:0_	18.93	22.34	18.74	17.97	21.18	17.11
	^2^C_16:1_	2.46	2.56	2.43	2.41	2.49	2.41
	^3^C_17:0_	0.51	0.51	0.49	0.49	0.50	0.47
	^4^C_17:1_	0.38	0.38	0.37	0.35	0.37	0.35
	^5^C_18:0_	13.05	13.37	12.91	12.61	13.06	12.35
	^6^C_18:1_	33.37	35.29	33.06	32.78	35.15	32.55
	^7^C_18:2_	14.09	16.41	13.80	13.57	16.07	13.28
	^8^C_18:3_	0.83	1.57	0.82	0.80	1.50	0.77
	^9^C_20:4_	6.32	6.33	6.29	6.21	6.31	6.15
fatty acid profile (%) in leg chicken nuggets	SFA	32.48	36.22	32.13	31.06	34.74	29.93
	MUFA	36.20	38.23	35.86	35.55	38.01	35.31
	PUFA	21.24	24.31	20.91	20.58	23.88	20.20
	UFA	57.43	62.53	56.77	56.13	61.88	55.51
	SFA/UFA	0.57	0.58	0.57	0.55	0.56	0.54
	PUFA/SFA	0.65	0.67	0.65	0.66	0.69	0.67

^1^ *Palmitic* ^*2*^ *Palmitoleic* ^*3*^ *Margaric* ^*4*^ *Margaroleic* ^*5*^ *Stearic* ^*6*^ *Oleic* ^*7*^ *Linoleic* ^*8*^ *Linolenic* ^*9*^ *Arachidonic*

Control, WGO = (wheat germ oil (Natural α-tocopherol) 200 mg/ kg feed), AT (α-tocopherol 200 mg/kg feed), ALA = α-Lipoic acid 150 mg/kg feed, WGO + ALA = wheat germ oil (Natural α-tocopherol) 200 mg/ kg feed) + α-Lipoic acid 150 mg, ALA + AT = α-Lipoic acid 150 mg + α-tocopherol 200 mg/ kg feed

*SFA* Saturated fatty acids, *MUFA* Mono unsaturated fatty acids, *PUFA* Poly unsaturated fatty acids, *UFA* Unsaturated fatty acids, *SFA/UFA* Ratio of saturated fatty acids and unsaturated fatty acids, *PUFA/SFA* Ratio of poly unsaturated fatty acids and saturated fatty acids

### Sensory evaluation of chicken nuggets during storage

**Fig. 3:**
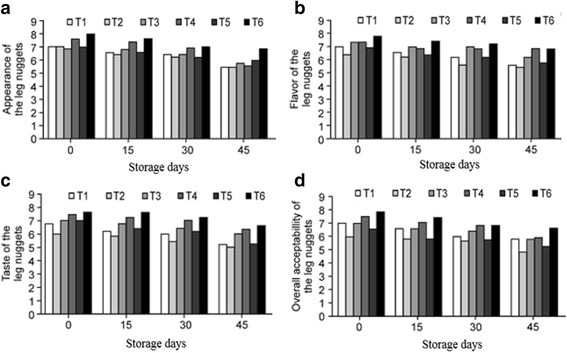
(**a-d**): (**a**) Appearance of the nuggets (**b**) Flavor of the nuggets (**c**) Taste of the nuggets (**d**) Overall acceptability of the nuggets

The results of the sensory analysis of attributes like appearance, flavor, taste, and overall acceptability of the chicken nuggets are given in Fig. 3. The sensory scores given by the panel of judges to appearance, flavor, taste and overall acceptability varied significantly between the nuggets from the different diet groups and storage intervals. The results showed that chicken nuggets made from chickens that were fed the combination of AT and ALA scored higher for all attributes on the 45th day of storage whereas the nuggets made from WGO-fed chickens scored lower but within the acceptable limits.

It is evident from Fig. 3 that the sensory score given by the panel of judges decreased as the storage interval increased. This result is in agreement with the results of many other researchers; the appearance and color of different meat products diminished as the storage interval increased [55, 56]. Other researchers have reported that the panel of judges had a better sensory perception of the nuggets enriched in natural antioxidants [57]. The score for flavor also decreased with storage due to the loss of volatile flavoring compounds during storage. This hypothesis was supported by Thomas et al. [58] and Bhat et al. [59]. The overall acceptability also decreased during storage because of the decline in the sensory score of other parameters like appearance, flavor, and taste. This decrease in overall acceptability was confirmed by the results of Malav et al. [60] who reported that the overall acceptability of mutton patties decreased during storage.

## Conclusions

It is concluded that the chicken nuggets made from the leg meat of chickens fed with diet supplemented with both WGO and ALA have better antioxidant potential as well as stability during storage. Nuggets from the group of animals fed with WGO supplemented diet showed better fatty acid profile because of the higher PUFA content in WGO. It is believed that ALA regenerates other antioxidants like AT and ascorbic acid. Therefore, nuggets from the group fed with diet containing WGO + ALA had higher content of ALA and AT because of the synergism between ALA and AT. Regarding the descriptive sensory evaluation, the overall acceptability of chicken nuggets from the animals fed with both ALA and AT was the highest according to the scores awarded by the panelists. However, this score decreased on storage.
